# Suicidal ideations, plans and attempts in primary care: cross-sectional study of consultants at primary health care system in Morocco

**DOI:** 10.11604/pamj.2016.24.274.9060

**Published:** 2016-07-27

**Authors:** Bouchra Oneib, Maria Sabir, Yassine Otheman, Naima Abda, Abderrazzak Ouanass

**Affiliations:** 1 Department Psychiatry, Faculty of Medicine, University Mohammed I, Oujda Morocco; 2 Department of Psychiatry, Faculty of Medicine, University Mohammed V, Rabat, Morocco; 3 University Psychiatry Center El Hassan, Faculty of Medicine University Sidi Mohammed Ben Abdellah, Fès, Morocco; 4 Laboratory of Epidemiology, Faculty of Medicine University Mohammed I, Oujda, Morocco

**Keywords:** Suicidal ideation, suicidal risk, primary health care, Morocco

## Abstract

**Introduction:**

The aim of the study is to estimate the prevalence of suicidal ideation among Moroccan consultants in primary health care system.

**Methods:**

We conducted a cross sectional survey in three health care centers in two cities of Morocco to estimate the prevalence of suicidal ideation, plan and suicide attempts among 396 consultants in the primary health care system, using the Mini International neuropsychiatric interview. Patients were 18 years and older, without known psychiatric or chronic somatic disease. Statistical analysis was performed by the SPSS 13.0 software.

**Results:**

The prevalence of suicidal ideation was 5.3%, and 2.7% of the patients planned their suicide and 1.2% tried to commit suicide. The multivariate analysis did not demonstrate significant association.

**Conclusion:**

Suicidal ideation, plan and suicide attempts are prevalent in primary health care patients, but they are still under diagnosed. An adequate training of physicians and the establishment of education programs is essential to reduce the rate of suicide.

## Introduction

Suicide is a major public health problem in several countries. More 1 million deaths worldwide per year were due to suicide [1]. Depressive disorders are the most involved in suicidal risk [2]. In fact, depressed patients had a suicide risk 20 times greater than the general population [3]. Early detection of suicide ideations allows effective prevention [4]. We aimed here to determine the prevalence of suicidal ideations plans and attempts, among primary health care consultants.

## Methods

Study sample and procedure: it is a cross sectional study performed in three health care centers in two cities of Morocco to estimate among others, the prevalence of suicidal ideation among the consultants in primary health care. The sample size was calculated according to the prevalence of suicidal ideation by using the following formula:

N= 1,96^2^ p. (1- p)/ margin error^2^

With: p = the expected proportion of subjects with suicidal ideation. Margin error: is between 0,03^2^ and 0.05^2^

The population of the study was selected from the healthcare users aged 18 years old or more. Individuals treated for a psychiatric disorder and / or chronic and disabling physical illness such as diabetes, endocrine disorders, neurological disorders, cancer and others were excluded. We also excluded patients who have not given their verbal consent. The ethical permission was obtained from the Ethical Committee of Biomedical Research University Mohammed V Rabat. Information was collected between August 2012 and December 2014 and it was done by psychiatrist and physicians who received a psychiatric training for the MINI. It was conducted using a standardized questionnaire with two parts. The first part assesses the demographic characteristics of consultants: age, gender, marital status, level of education and socioeconomic level of the participants. The second part used the Mini International neuropsychiatric interview (MINI), to assess suicidal ideations, plans and suicide attempts. In accordance with the MINI, we considered patients having suicidal ideation when they had, during the last four weeks, thoughts of death, suicide, suicide plan or when they tried to commit suicide. The MINI has been translated and validated in Moroccan dialect; the validity study demonstrated a satisfactory quantitative assessment of the value of kappa> 0.8 [5]. The time spent to evaluate each participant by the questionnaire was 30 min.

Statistical analysis: statistical analysis was performed by the SPSS 13.0 software (Statistical Package for the Social Sciences). A descriptive analysis of the different parameters was made. Quantitative variables were described as a means ± standard deviation and categorical variables were expressed as percentages. A search for factors associated with suicidal ideations was made using the chi 2 test or t student. Then logistic regression was used to take into account possible confounding factor. A p <0.05 was considered significant.

## Results

The sample consisted of 396 patients, almost two-thirds were women; the demographic characteristics are detailed in Table 1. The prevalence of suicidal ideation during the last month was 5.3%, and 2.7% of the patients planned their suicide, meanwhile 1.2% tried to commit suicide. According to the MINI interview, 6.8% met the criteria of suicidal average risk and 6.8 % had a higher suicide risk. In the univariate model, we found that suicidal ideation were associated with female gender and unemployment. The multivariate analysis and adjustment of the OR has not resulted in a significant association (Table 2).

**Table 1:** Socio-demographic characteristic

The characteristics	N (%)	patients with suicide risk
Age (years)^+^	39, 9 ±15^+^	45.7 ±17^+^
**Gender**		
Man	146(36,9)	2(9.5)
Women	250(63,1)	19(90.5)
**Profession**		
With	123 (31,1)	2(9.5)
Without	273 (68,9)	19(90.5)
**Marital status**		
Live alone	151(38,1)	7(33.3)
Couple	245(61,9)	14(66.66)
**Level of education**		
Schooled	82(20,7)	15(71.4)
Not schooled	314(79,3)	6(28.6)
**Socioeconomic level**		
Low	175( 44,2)	10(47.6)
Medium	221(55,8)	11(52.4)

Means± Standard deviation

**Table 2:** Univariate and multivariate analysis

Suicidal ideation p OR (95% IC)		
	Univariate	Multivariate
Gender	0.01 6[1.3, 26]	0.2 3.4 [0.5, 23]
Profession	0,04 0.2 [0.05, 0.9]	0.7 0.7 [0.09, 5.8]

## Discussion

Suicide risk is usually associated with psychiatric disorders, especially depressive ones [6, 7]. Psychiatrists are usually trained to evaluate and manage this risk. However, suicidal behaviour and ideations remains under-detected in the primary health care system [8, 9]. Our study is in our knowledge, the first one in Morocco, which estimates the suicidal thoughts within the primary health care system. Our results are similar to those of other studies in which the rate of suicidal ideation was between 2% and 7% of all primary care patients [10, 11] (Table 3). Epidemiological studies led in general community or in primary health care, about suicidal behaviours showed different prevalence’s of suicidal ideation, plan and suicide attempts. That can be explained by the use of different instruments and methodological procedures, but also by socio-cultural specificities. In our study, patients with known psychiatric or chronic somatic disorders were excluded because they were usually seen by specialists, and the suicidal risk is higher and known for these patients [12, 13]. We did not find a significant association between suicidal ideations and socio-demographic characteristic in multivariate analysis, however, other studies demonstrated that the suicide risk is associated with male gender, alcohol abuse, a family history of psychiatric disorder and comorbidity with other psychiatric disorders [14, 15]. To prevent suicide risk in primary health care, physicians need to look for suicidal ideation. The majority of patients who committed suicide were in close contact with general physicians in primary health care before their death [16, 17]. The studies demonstrated that 40% of these patients have seen their physician in the three months before the death, and 20% in the week, that preceded the suicide [18–20]. Establishing a screening program for mood disorders and suicidal risk may reduce the prevalence of suicide [21]. Some studies have shown that therapeutic education programs among physicians increased the prescription of antidepressant and reduced the rate of suicide [22, 23]. The limitation of our study is the relatively small sample size, which may explain the difficulty to identify predictors’ factors.

**Table 3:** Summary of some study of suicidal ideation, plan and attempts suicide out coming in primary health care and general population

Authors (place)	Results (prevalence)		
	Suicidal ideation	Plan	Attempts
Bunevicusetal., (Lutenia)	6%	-	-
Ono Y et al., (Japan)	10.9%	2.1%	1.9%
Kebede D et al., (Ethiopia)	2.7%	-	0.9%
Kessler et al., (USA)	13.5%	3.9%	4.6%
Malakouti SK et al., (China)	5.7 %	2.9%	1%
Botega et al., (Portugal)	5.3%	4.8 %	2.8%
Weissman et al., (9 countries)	2.1% 18.5%	- -	0.72% 5.93%
Beautrais et al., (New Zeland)	3.2%	1%	0.4%
Scocco et al., (Italy)	3%	0.7%	0.5%
Kjøller et al., (Danmark)	6.9%	3.4%	0.5%
Gabilondo et al.,(Spanish)	4.4%	-	1.5%

## Conclusion

Suicidal ideations are common in primary health care, however, they still under-diagnosed. An adequate training of the physicians and establishing educational programs is essential to reduce the rate of suicide in this population.

### What is known about this topic

- Other studies had demonstrated that suicidal ideations were common in primary health care.

### What this study adds

- This is the first study conducted in primary health care in Morocco to estimate the prevalence of suicidal ideation;
- The study estimated also the prevalence of suicide plans and attempts.
